# Oral Pre-Exposure Prophylaxis (PrEP) Awareness and Acceptability Among Persons who Inject Drugs (PWID) in Kenya: A Qualitative Investigation

**DOI:** 10.21203/rs.3.rs-2512731/v1

**Published:** 2023-02-22

**Authors:** Mugambi Cosmas, Mbogo Loice, Sinkele William, Gitau Esther, Farquhar Carey, Temu Tecla, Bukusi David, Kinuthia John, Monroe-Wise Aliza

**Affiliations:** Ministry of Health; University of Washington, School of Global Health; Support for Addictions Prevention and Treatment in Africa (SAPTA); Support for Addictions Prevention and Treatment in Africa (SAPTA); University of Washington, School of Global Health; University of Washington, School of Global Health; Kenyatta National Hospital; Kenyatta National Hospital; University of Washington, School of Global Health

**Keywords:** People who inject drugs, Pre-Exposure Prophylaxis, awareness, uptake, Human Immunodeficiency Virus, Barriers, Uptake

## Abstract

**Background:**

People who inject drugs (PWID) are disproportionately affected by HIV despite the availability of multiple efficacious biomedical prevention interventions including oral pre-exposure prophylaxis (PrEP). Little is known about the knowledge, acceptability, and uptake of oral PrEP among this population in Kenya. To inform the development of oral PrEP uptake optimization interventions for PWID in Kenya, we conducted a qualitative assessment to establish oral PrEP awareness and willingness to take PrEP by this group in Nairobi City.

**Methodology:**

Guided by the Capability, Opportunity, Motivation, and Behaviour (COM-B) model of health behavior change, we conducted 8 focus group discussions (FGDs) among randomly constituted samples of PWID in four harm reduction drop-in centers (DICs) in Nairobi in January 2022. The domains explored were: perceived risks (behaviour), oral PrEP awareness and knowledge (capability), motivation to use oral PrEP (behaviour), and perceptions on community uptake (motivation and opportunity). Completed FGD transcripts were uploaded to Atlas.ti version 9 and thematic analysis was conducted through an iterative process of review and discussion by two coders.

**Findings:**

There was a low level of oral PrEP awareness with only 4 of the 46 PWID having heard of PrEP; only 3 out of 46 participants had ever used oral PrEP and 2 out of 3 were no longer using it, indicating a low capacity to make decisions on oral PrEP. Most study participants were aware of the risk posed by unsafe drug injection and expressed willingness to take oral PrEP. Nearly all participants demonstrated low understanding of the role oral PrEP plays in complementing condoms in HIV prevention, presenting an opportunity for awareness creation. While the PWID were eager to learn more about oral PrEP, they favored DICs as places where they would like to obtain information and oral PrEP if they chose to use it, identifying an opportunity for oral PrEP programming interventions.

**Conclusion:**

Creation of oral PrEP awareness among PWID in Kenya is likely to improve uptake since the PWID are receptive. Oral PrEP should be offered as part of combination prevention approaches, and effective messaging through DICs, integrated outreaches, and social networks are recommended to mitigate displacement of other prevention and harm reduction practices by this population.

**Trial Registration:**

ClinicalTrials.gov Protocol Record STUDY0001370.

## Background

The target to end the 40-year HIV/AIDS epidemic by the year 2030. ^[1]^ However, the persistent threat of HIV and AIDS amid ongoing stigma has cast doubt that 2030 is a realistic goal. ^[2]^ Oral PrEP and post-exposure prophylaxis have proven to be effective strategies, but gaps in HIV prevention still exist.^[3, 4, 5]^ In sub-Saharan Africa, where oral PrEP continues to roll out at scale, coverage is patchy, with uptake greatest in Kenya and South Africa.^[6]^

Kenya and Tanzania are ranked fourth in terms of the national HIV burden which is estimated at 1.6 million in both countries. ^[7]^ In Kenya, HIV is largely spread through the sexual route and one in every three new HIV infections are attributed to key population groups, including men who have sex with other men (MSM), sex workers and PWID. ^[8, 9]^ People who inject drugs (PWID) in Kenya have an estimated HIV prevalence of 18.3%, compared with the prevalence 4.9% in the general population. ^[11, 12, 67]^ Although Kenya has succeeded in scaling up harm reduction programs among key populations, prevention of new infections among key population groups remains challenging.^[13, 14]^ Therefore, identifying effective strategies to prevent HIV among PWID and their partners is central to overall HIV prevention efforts in Kenya. ^[15]^

Oral PrEP has been shown to reduce the risk of HIV infection from unprotected sex by over 90%, and from injecting drugs by more than 70%.^[16]^ Oral PrEP is thus being scaled up as an important intervention for controlling the HIV epidemic when combined with other strategies such as consistent condom use, risk reduction counseling, and HIV testing.^[17]^ For this reason, in November 2015, the World Health Organization (WHO) recommended that all people at substantial risk of acquiring HIV infection be given the opportunity to take a daily antiretroviral pill for oral PrEP against HIV, a recommendation that is also documented in the framework for the Implementation of Pre-Exposure Prophylaxis of HIV In Kenya. ^[13, 18, 19]^

However, despite the documented efficacy of oral PrEP, strategies for optimal scale-up and adherence remain unclear and the best oral PrEP delivery models in generalized epidemics are not known.^[20, 21, 22, 23, 24, 25]^ Adherence to oral PrEP use and retention in oral PrEP services range from 3.0–35.0% and are especially low in low- and middle-income countries. ^[26, 27, 28]^ With limited studies on the barriers to oral PrEP uptake, adherence, and retention among key populations, it is believed that the same factors impending HIV treatment are to blame for the challenges in oral PrEP use.^[29, 30]^ Ongoing national PrEP programs in Africa have capitalized on awareness and demand creation through mass media and community health care workers.^[31]^ In the sub-Saharan Africa context, challenges in PrEP programming persist, including the need to improve PrEP access, optimal delivery models, training of health care workers, and retention into oral PrEP care.^[32]^ For example, despite oral PrEP tablets collection by men who have sex with men (MSM) in Kenya, retention, and adherence to the same is low.^[33]^ Thus, a scale-up of oral PrEP use among PWID guided by an understanding of specific barriers faced by this group is therefore required.^[23, 34]^

To achieve sustainable control of HIV transmission among PWID and their partners, evidence-based HIV prevention strategies are required and this should be informed by existing service challenges and gaps. ^[35, 36]^ For this reason, there is need to evaluate oral PrEP awareness and acceptability among PWID and their injecting and sexual partners in Kenya. ^[37, 38]^ Additionally, key barriers such as user challenges, access, and adherence and effectiveness among PWID and their partners must be evaluated with an objective of adapting best delivery models, making social and behavioral impact and integration of oral PrEP services with other services targeting these population groups. ^[37, 38]^ Whereas oral PrEP has been scaled up in Krenya’s general population, gaps still exist among KPs including PWID.^[39, 40]^ In light of these contextual gaps, this study was aimed at investigating oral PrEP awareness and acceptability among PWID in Nairobi County to inform the development of oral PrEP interventions for PWID and their partners.

## Methods

**Figure 1:**
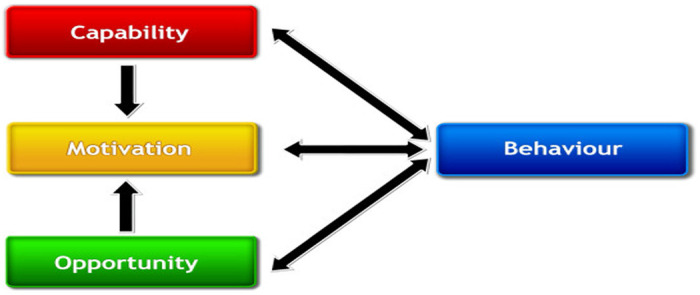
COM-B model ^[65,66]^

We used a theory-based model to understand oral PrEP awareness and acceptability among PWID in Nairobi with the objective of developing tailored interventions to promote oral PrEP uptake and adherence. As part of the formative process, we conducted a baseline assessment through focus group discussions (FGDs) whose development and analysis were guided by the Capability, Opportunity, Motivation and Behaviour (COM-B) model.^[65, 66]^ In brief, the COM-B model asserts that capability, opportunity, and motivation interact to generate behaviour. The single-headed and double-headed arrows in Fig. 1 below represent potential influence between components in the system. The causal links within the system can work to reduce or amplify the effect of particular interventions by leading to changes. In our case, we were interested in what components of the behaviour system would need to be changed to achieve oral PrEP uptake (Fig. 1).

In January 2022 we conducted 8 FGDs among randomly constituted samples of HIV-negative PWID in Nairobi county recruited through peer educators (program staff providing day to day outreaches and awareness sessions on the harms of drugs injection, safe needle and syringe use and HIV infection prevention) working in four DICs through word of mouth. Eligible PWIDs were recruited to participate in the 8 FGDs that were stratified by gender (one male and one female FGDs in each of the four drop-in centers). The overall goal was to assess oral PrEP awareness, experiences, acceptability, and strategies for optimizing uptake in this population. The FGDs were conducted by two female qualitative interviewers (both holding master’s degrees in public health) who have previously worked with PWID and other KPs and interviews took place in DICs which are considered safe spaces for PWIDs and privacy was maintained.

Study inclusion criteria were age 18 years and above, self-identification as PWID, active injection in the preceding six months, no self-reported acute HIV infection or established HIV infection, and ability to consent to participation in the study.

Guided by the COM-B framework, we designed a semi-structured focus group interview guide to document oral PrEP awareness and acceptability among the PWID. The domains explored were: perceived risky behaviours which denote motivation under the COM-B framework, oral PrEP awareness, and knowledge which denote capability and opportunity under the COM-B model, and motivation to use oral PrEP and perceptions on community uptake which signify motivation and opportunity to take up oral PrEP under the COM-B model.

FGD sessions lasted 80 to 100 minutes and were conducted in Swahili, audio-recorded then transcribed and translated to English. Each FGD participant was reimbursed transport costs at a uniform rate of 400 Kenya Shillings (4 USD). Written informed consent (Swahili and English versions) was given by all the FGD participants.

Completed FGD transcripts were uploaded to Atlas.ti version 9 for analysis. We selected 20% of transcripts to check for accuracy in comparison with their respective Swahili audio-files. An initial codebook was developed by two coders based on a subset of transcripts and literature review. Additional codes were those that emerged during the coding process and were included in the final codebook. Using the final codebook, all transcripts were coded by one coder and then coding reviewed by a second coder. Disagreements in coding were resolved through discussion. Using thematic analysis, themes were arrived at through an iterative process of review and discussion by the two coders.

Institutional Review Board (IRB) approval was obtained from the University of Washington’s Human Subjects Division and the Kenyatta National Hospital (KNH)/University of Nairobi (UON) Ethical Review Committee, and a research permit was obtained from the National Commission for Science, Technology and Innovation (NACOSTI) in Kenya. All methods were performed in accordance with the relevant IRB guidelines and regulations.

## Results

A total of 46 PWID participated in the eight FGDs in the four DICs (52.2% males and 47.3% females, Table 1). Most participants had a primary school level of education (82.4%) with only 17.6% having secondary school education level. In terms of livelihoods, 47.1% were casual labourers, 32.4% were engaged in sex work and 8.8% were self-employed while 11.8% had no source of income. The average monthly income was Kenya Shillings 0 to 5000 (0-50 USD) per month for 41.2% of the FGDs participants, KSHs 5000 to 10,000 (50-100 USD) per month for 47.1% of them and KSHs 10,000 to 20,000 (100-200 USD) per month for the remaining 11.8% (Table 1).

### Oral PrEP Awareness and Knowledge Levels (Capability)

The majority of participants (93.5%) in this study had never heard of oral PrEP. They were excited to learn about oral PrEP as expressed by the quote below. It was evident that this high-risk HIV group had limited access to health information and services since they largely lived in hideouts due to fears of stigma and discrimination, fear of law enforcement agencies, inconsistent access to information sources and unavailability of differentiated services targeting the group.

*“I also had no idea of what PrEP is, I have heard about it today and I am happy I will know more about it from this forum. It will help us and we will help others.”[*FGD 1 participant, Male]

It was evident that even after providing brief information on oral PrEP, some of the participants still did not understand basic aspects prompting facilitators to revisit the informational poster during the discussion. This low comprehension of the provided information reflects the low education levels among the PWID as illustrated in Table 1 and is also indicative of the need for simplified messages on PrEP targeting PWID.

*“I have learned about PEP and PrEP today, just now. That’s why you can see I have been silent because I don’t understand.” [*FGD 2 participant, Female]

A few participants reported that they had heard about or used oral PrEP but were confusing post-exposure prophylaxis (PEP) with oral PrEP. Given that awareness largely spread through networks, the confusion between PrEP and PEP indicates a lack of clarity between these two HIV prevention measures, demonstrating the need for targeted education and prevention efforts for PWID. Related to the aforementioned is the fact that, in Kenya, PEP was rolled out before PrEP hence a higher level of PEP awareness among key populations.

***“**Even when you are raped, you can also use PrEP.”* [FGD 4 participant, Female]

*“I have ever used it, when I was injecting, I noticed that the cotton wool that I was using had stains of human blood but since it was at night nothing much I would have done so I went to the hospital and was prescribed PrEP which I used for 28 days after which I went for a blood test which came out negative.”* [FGD 3 participant, Male]

While some of the participants had heard of oral PrEP, they did not have any understanding of what it entailed and desired to gain understanding. This observation points to substantial unmet information needs due to the limited information available to the PWID.

*“I have ever heard of PEP and PrEP but have never known the difference so it’s good that you are here to expound more on it.”* [FGD 6 participant, Male]

Given the lack of information on oral PrEP in this population and the enthusiasm to learn more, PWID had several questions about oral PrEP. Specifically, eligibility criteria for PrEP use, use of PrEP concurrently with other medications, and places where the oral PrEP tablets could be obtained were enquiries from the FGD participants.

***“**Just asking. If I am HIV positive, can PrEP be helpful?”* [FGD 7 participant, Male]

*“Let’s say you were taking those PrEP drugs and you are also taking other drugs; can that be a problem?”* [FGD 8 participant, Female]

*“My question is, where can PrEP be found currently?”* [FGD 7 participant, Male]

It was evident that participants had misconceptions on how to correctly use oral PrEP and the timing and duration of taking PrEP drugs. They perceived that oral PrEP was to be used right before a sexual encounter. Misinformation about this novel HIV preventative measure was evident and this has implications on impeding PrEP uptake. Therefore, the likely misinformation proliferation through shared opinions among peers featured strongly among participants.

*“Before we thought you take PrEP right before sex, we didn’t know how long to use it, or when to start. So, we have been enlightened. We could have made a mistake. But now we have been helped.”* [FGD 4 participant, Female]

*“I knew about it, what I didn’t know is for how many hours you should take the drugs prior to having sex with your partner so that you cannot be at risk of getting infected. I didn’t know if it’s 24 hours or 20 hours.” [*FGD 1 participant, Male]

There were varied opinions on oral PrEP and condom use with some participants advocating for combined use while others expressing that either oral PrEP or condom alone should suffice. These mixed opinions indicate low awareness that PrEP only prevents a person against HIV transmission and the recommended use of PrEP is in conjunction with condoms to both maximize effectiveness and prevent against other sexually transmitted infections.

*“There is no need for condom use because the purpose of using PrEP is basically for protection against the virus, so if you are negative and you are using PrEP, you can comfortably have sex with the person who is positive without any worry.” [FGD 3 participant, Male]*

***“**I feel like you can still go ahead and use a condom because they all serve the same purpose of protection against the virus.” [FGD 6 participant, Female]*

*“Maybe they are on PrEP and are on family planning as well. So, they would feel that they are safe from HIV and pregnancy. And they also know that these other STIs are treatable”. [*FGD 5 participant, Female]

Participants further explained that female participants may be less likely to use condoms as they may be using other methods to prevent pregnancy and in case they contract STIs, they are confident to receive treatment. Whereas STIs are one of the potential outcomes of sex that one would like to avoid, availablity of treatment for STIs and the knowledge that these infections were curable promoted unsafe sex in the group. Therefore, it was evident that interrupting transmission of infection, preventing re-infection and treating sexual partners was not only difficult in this group but also poorly understood. These perceptions may pose serious obstacles to STIs prevention efforts due to their influence on social and sexual networks, access to and provision of care, willingness to seek care, and social norms regarding sex and sexuality. Misconceptions about STI risk and treatment necessitate accurate risk assessment and education and counseling regarding STI prevention through changes in sexual behaviors and use of the recommended prevention services. Recommended PrEP regimens do not appear to alter the effectiveness of hormonal contraceptives and the FGD participants noted that use of PrEP and contraceptives is likely to increase the dual protection from HIV and unwanted pregnancies.

“I think they should go hand in hand. I think PrEP can be used together with a condom.” [FGD 7 participant, Male]

### Willingness to use Oral PrEP (Behaviour)

Participants in this study expressed interest in using oral PrEP, driven by the perception of high risk for HIV due to sharing needles and equipment. Others added that they felt the need to protect themselves against HIV as they were at high risk due to engaging in sex work. This self-perception of being at high risk presents an opportunity for PrEP awareness creation and promotion among PWID.

*“I would love to use PrEP because like my friend has said, we inject drugs and as I had said earlier now that there is a shortage of needles, it puts us on a very high risk. With the idea of PrEP, I would have started using it earlier if I knew of it but now that I know it, I will begin using it.”* [FGD 1 participant, Male]

*“If you’d have a job, at least you can get money, but since you don’t have a job, now you have to ‘commit sin’to make ends meet. That’s why sometimes people choose to use such things [PrEP]”. [*FGD 8 participant, Female]

However, there were also a few participants who were not keen on using PrEP but were glad they were equipped with information to be able to decide if they got to a point they felt they needed PrEP. The quote below indicates that some PWID were happy to have learnt about PrEP but were not yet convinced to begin using it.

*“I am glad I have learned about it but I can’t say I want it. I have not decided yet about it, but I am glad to have learned about it.”* [FGD 5 participant, Female]

### Optimal PrEP Delivery (Motivation and Opportunity)

Drop-in centers (DICs) are preferred as a source of information on oral PrEP and oral PrEP access. The participants mentioned other places such as government hospitals where their wider community can access oral PrEP and oral PrEP information but DICs were preferred over public facilities. Participants argued that it may be difficult for them to access information through other sources or media. DICs were preferred because of the privacy and safety attributed to these sites as well as the differentiated care and attention offered to the participants. Access to information from media and other community members was equally low due to economic barriers and social and security concerns that restrict their interactions with the general population.

*“It may be difficult doing sensitization, it will depend on personal will but for us drug addicts, most of us come to DICs, so for us it will be easier for us here at the DIC. Speaking about us who engage in substance abuse, it’s difficult to find someone watching TV or reading a newspaper.”* [FGD 7 participant, Male]

*“I prefer DICs because most of us have no time to listen to radio or television and therefore almost 90% of us can gather first-hand information from a DIC because it is where we even come to take our lunch, furthermore there is specific information that one would want to hear and is not discussed either in radio or television so they are not reliable sources to get the information.”* [FGD 3 participant, Male]

They also noted that it would be beneficial to have sensitization sessions on oral PrEP integrated in needle and syringe programs and targeted outreach programmes. This preference once again highlights privacy concerns and differentiated approaches for PrEP promotion in this population.

*“I was thinking that those that come for the outreach to bring needles, could gather the men and talk to them. Not just bringing the needles and just leaving. At least talk to the men before they leave. Not everyone can afford the fare to come here, so, those that come to bring the needles could come with the kits for testing and then give the information [on PrEP]..” [FGD 8 participant, Female]*

Participants also believed that oral PrEP could be beneficial if it was offered closer to them in the community. The quote below highlights the aforementioned confusion of PrEP for PEP. At the same time the aspect of travel distance to access PrEP is presented through preference for communal points for PrEP distribution and this may be attributed to privacy concerns and economic inaccessibility.

*“Another thing, during sex not many people will be honest. After the sex is when you will come to realize you have been infected so oral PrEP will be good if they are available at the places we hang out. In case there comes someone to give out PrEP, it will be so good”.* [FGD 7 participant, Male]

Oral PrEP availability will also enhance information reaching the intended population. Practical teachings with demonstrations as well as showcasing oral PrEP tablets would help clear some of the myths and misconceptions that PWID hold and also offer assurance that these drugs can be locally availed to those in need.

*“One way to raise oral PrEP awareness is by teaching them and making PrEP available close to them. That will be one of the easier ways”* [FGD 7 participant, Male]

In order to encourage uptake, messages on oral PrEP should besimple and should leverage lived experience to demonstrate that those who have used PrEP have had positive experiences. The messages should focus on the role of oral PrEP, how it is to be used and by whom, and the benefits. The messages can also include where they can access oral PrEP and the possible side effects. When counselling PWIDs on oral PrEP, simple messages on benefits can motivate uptake and providers should use relatable examples. The quotes below indicate that the long-term protection of PWID from HIV can demonstrate the effectiveness of this intervention. Simplification of messaging on oral PrEP would make it easy for the PWID population to understand given that most of them have low education level.

*“You first define for them what PrEP is then go ahead and discuss its importance, what exactly does it protect you from and you can as well demonstrate the difference between those who are using it and those that don’t”.* [FGD 6 participant, Male]

*“If you have a positive partner and using either PEP or PrEP and you’ve not been infected with the virus then you can as well share with others about it.”* [FGD 3 participant, Male]

*“You can also tell some of the side effects, and if someone encounters these side effects, how to deal with them”.* [FGD 4 participant, Female]

Participants mentioned the peers as another approach to raising awareness about and ensuring access to PrEP; however, challenges were also mentioned associated with stigma. They provided suggestions on how to be tactical when using this model. Once again, being accompanied by peers for PrEP refills not only highlights the peer to peer model but also privacy and safety concerns that were persistently mentioned in the FGDs.

*“It’s even important if you ask any of your friends to accompany you to some of these centers to go pick the drugs[PrEP] because through this they will get to learn more information about PrEP and become good ambassadors”.* [FGD 3 participant, Male]

*“I guess you can just volunteer and say “I thought PrEP was to be used just when you want to have sex, on the contrary, it is to be used for a long time. Not just once. If it is used once, it can backfire”. You just tell them openly. So, if there was someone who didn’t know about it, they get to know..”* [FGD 4 participant, Female]

When asked how to support sustaining oral PrEP use among PWID, participants provided suggestions on a variety of options for where PWID can access the medication and with what frequency. Some of them felt picking PrEP daily at the DIC may be preferred while others felt it would be better to get a monthly dose. Some participants mentioned that living arrangements may have implications on PrEP uptake given that some of the PWID lived in the street while others had shared accommodation. Stigma from swallowing daily pills might therefore limit PrEP uptake among the PWID population.

*“For us who abuse drugs, if we are given the PrEP to take home, we are prone to ignoring things and someone can easily ignore taking their drugs. So it will be better to use the drugs from here.”* [FGD 2 participant, Female]

*“For the confidentiality purpose one might prefer the monthly one because of avoiding being seen attending the center on either weekly or daily.” [*FGD 3 participant, Male]

### Perceived Barriers to PrEP Use [Capability and Opportunity]

Three main perceived barriers to PrEP use were documented in the FGDs and these were the timing and frequency of swallowing PrEP, effects of missing out on daily doses, and accessing the distribution locations for PrEP pills. Mitigation of privacy-related barriers associated with daily oral PrEP use, the efficacy of PrEP, and ease in obtaining PrEP tablets and monthly refills would therefore support opportunities for uptake, retention, and adherence to this biomedical intervention.

**FGD moderator:** What can be the challenges to using PrEP?

**FGD participant: “**Is it meant to be used at a specific exact time?”

**FGD participant**: “Is there a place where we can go for it?”

**FGD participant**:“ May be forgetting”

**FGD participant:“** Is it wrong if I take the medication today and miss taking it tomorrow?”

**FGD participant:“** So I will have to use it each day?” [FGD 2 participants, Female]

## Discussion

Despite the high risk of HIV among PWID in Nairobi, their level of awareness, knowledge, and use of oral PrEP in 2022 is still surprisingly low: less than eight percent of participants knew about PrEP for HIV prevention. These findings align with the third National Behavioural Assessment of Key Populations in Kenya conducted four years ago (in 2018) which indicated that only 3.0% of the PWID in the country were using oral PrEP including 6.0% in Nairobi. ^[41]^ The low oral PrEP awareness levels indicate may be a reason for the suboptimal uptake of this preventive innovation.

Although the Kenya national guidelines have recommended PrEP for individuals at substantial risk of HIV infection, its use remains low; indeed, among adolescent girls and young women seeking family planning services in clinics in parts of Western Kenya with high HIV prevalence, only 4.0% had ever taken up oral PrEP.^[42, 43]^ Similarly, among women living with HIV in Kenya in 2018, only 6.0% were aware of PrEP as a method for protecting HIV-uninfected partners from acquiring HIV.^[44]^ Globally, low PrEP awareness and knowledge among PWID have been equally documented, with knowledge rates of 25% in Baltimore, 31.0% in New York, and 6.1% across twenty-two cities in India.^[45,46]^ Overall, there is low use of PrEP in this group in qualitative studies in Bangkok as well as quantitative studies in the United States with studies showing that ≤1% of PWID in n Seattle, Washington were taking oral PrEP in 2021.^[47,48]^ In addition, whereas PrEP is designed to be used in a planned way, on an ongoing basis and PEP is used in emergency situations, this distinction was not clear among the interviewed PWID. This finding has been documented among key populations in Portugal, Boston, Pittsburgh, San Juan and the northeastern region of the USA and it is attributed to low awareness of and exposure to these two interventions to these hidden population groups. ^[68, 69, 70]^

For all key populations, the World Health Organization (WHO) recommends offering oral PrEP to people at substantial risk of HIV infection, as part of combination prevention packages. The FGDs respondents have demonstrated a lack of clarity on the role oral PrEP plays as opposed to condom usage for HIV prevention.^[49]^ Oral PrEP users in Kenya have previously expressed their confusion and frustration with health care providers’ insistence on using condoms in addition to oral PrEP.^[50]^ Previous studies among key populations on oral PrEP in Kenya have, however, shown increases in condom use from 6/10 to 9/10 in three months when the correct information is provided.^[51]^ This calls for effective messaging to ensure the provision of oral PrEP while avoiding the displacement of existing condom use as guided by the World Health Organization.^[52]^

Upon provision of brief information regarding oral PrEP by the research teams, most PWID were receptive to this intervention due to the perceived high risk of HIV infection from unsafe injection practices, involvement in sex work and sex outside sex work given the high prevalence among peers and partners, all of which have been previously documented.^[53, 54]^ The perception of high HIV risk in this group of PWID is an opportunity for potential behaviour change interventions to minimize or eliminate HIV transmission risks. Globally, willingness to take up oral PrEP upon awareness creation has been shown due to the perceived risk of HIV. This may indicate that awareness creation combined with existing risk perception can increase uptake of oral PrEP.^[45]^ Multiple studies in USA have shown that increased awareness of PrEP was associated with higher willingness to use PrEP hence the need to ensure that PWID are knowledgeable about these pills.^[55,56, 57]^ Quantitatively, across various set ups including Canada and the United States, on average one in every three PWID expressed willingness to use PrEP.^[56,58]^

The interviewed PWID indicated a preference of DICs for oral PrEP information and access, a finding that has been previously been documented among adolescents in Kenya who opted for the safe space model due to the privacy that comes with such settings.^[59]^ This preference of DICs for information access offers an opportunity to provide behaviour change communication targeting PrEP uptake. As of 2017, 72% of the PWID in Kenya had visited a DIC including 75% of those in Nairobi, a further affirmation that these sites are popular among members of this vulnerable group.^[60]^ In the country, PWIDs have cited financial barriers to making visits to health centers, long distance to clinic and stigma as some of the reasons why they prefer DICs to health facilities for HIV services.^[61]^ In other settings, negative experiences with healthcare providers, stigma within social networks, poor infrastructure and low capacity for PrEP delivery in health facilities have been proven to be barriers to PrEP uptake among PWID. ^[56]^ Lastly, sensitization sessions on oral PrEP integrated in DICs and outreach activities have also been reported by these FGD participants as a suitable platform to create awareness on oral PrEP. The recommended use of outreach activities conducted by NSPs where peers can sensitize clients in the community presents an opportunity for oral PrEP awareness creation. Social networks that have the potential to motivate PWIDs can also be leveraged to increase uptake of oral PrEP, promote adherence and improve retention in this vulnerable population. This finding indicates that social networks and peer led outreaches within the larger NSPs may be efficient means for disseminating messaging about oral PrEP.^[62, 63,64]^

### Limitations and Strengths of the Study

This study was conducted among PWID living in Nairobi, limiting the generalizability of the findings. Similarly, the FGD participants were affiliated with DICs thus the perspectives of those with no exposure to DICs are not reflected in the study. Given that no other qualitative or quantitative study has explored oral PrEP dynamics among PWID in Kenya, the study has provided preliminary insights into this subject.

## Conclusion

This study has indicated a low exposure of PWID in Nairobi to oral PrEP interventions hence low awareness of the same which points out to low PrEP interventions targeting this group. This could be interpreted to affirm that PWID have specific lived experienced contexts and needs, and are burdened by social and economic marginalization and inequality in Nairobi City. Despite having inadequate knowledge about PrEP, study participants were largely willing to use PrEP if available due to the foreseen HIV risks. Strategies to raise awareness of PrEP, differentiate between PEP and PrEP, and improve their accessibility among PWID in Nairobi are needed. Improving awareness of PrEP through increasing access to PrEP-related health education and enhancing risk perceptions of HIV infection could have further positive effects on PrEP uptake among PWID. Programs should include effective information, education, and communication component around their preferences and provide PrEP in friendly sites. Specifically, existing DICs are recommended to provide PrEP-related interventions and so has tapping into the existing social networks. Lastly, further efforts are needed to understand perceptions of risk for HIV, determinants of PrEP use, and to investigate successful strategies for PrEP implementation and delivery in this marginalized population.

## Figures and Tables

**Table 1:** Social demographic background of the 24 male and 22 female FGD participants

Variable	Description	Male	Female	Total
**Gender**	DIC 1	21.1%	30.0%	29.4%
	DIC 2	26.3%	33.3%	29.4%
	DIC 3	26.3%	20.0%	23.5%
	DIC 4	26.3%	16.7%	17.6%
	**Total**	52.2%	47.8%	100.0%
**Education**	Primary school	84.2%	80.0%	82.4%
	Secondary school	15.8%	20.0%	17.6%
**Employment status**	Casual labour	36.8%	60.0%	47.1%
	Self employed	0.0%	20.0%	8.8%
	Sex Worker	0.0%	57.9%	32.4%
	Unemployed/None	5.3%	20.0%	11.8%
**Monthly income**	0-5,000 KSHs (0-50 USD)	31.6%	53.3%	41.2%
	5,001-10,000 KSHs (>50 USD to 100 USD)	63.2%	26.7%	47.1%
	10,001-20,000 KSHs (>100 USD to 200 USD0	5.3%	20.0%	11.8%

## Data Availability

All the data collection tools and data are in the custody of Dr. Cosmas Mugambi and are available on request.

## Abbreviations

AIDS: Acquired Immune Disease Syndrome
COM-B: Capability, Opportunity, Motivation, and Behaviour
DIC: Drop-in Center
FDA: Food and Drug Administration
FGD: Focus group Discussion
FSW: Female Sex Workers
HIV: Human Immodeficiency Virus
IRB: Institutional Review Board
KNH: Kenyatta National Hospital
MSM: Men who have Sex with Men
NACOSTI: National Commission For Science, Technology, and Innovation
NIDA: National Institute on Drug Abuse
NSP: Needle and Syringe Exchange Program
PPB: Pharmacy and Poisons Board
PrEP: Pre-Exposure Prophylaxis
PWID: Persons who Inject Drugs
SAPTA: Support for Addictions Prevention and Treatment in Africa
UNAIDS: United Nations Programme on HIV and AIDS
UON: University of Nairobi
USA: United States of America
USD: United States Dollar
WHO: World Health Organization
